# Symptomatic hypocalcemia in an epileptic child treated with valproic acid plus lamotrigine: a case report

**DOI:** 10.4076/1757-1626-2-7394

**Published:** 2009-06-17

**Authors:** Andrea Domenico Praticò, Piero Pavone, Maria Grazia Scuderi, Giovanni Li Volti, Renato Bernardini, Giuseppina Cantarella, Lorenzo Pavone

**Affiliations:** 1 Department of Pediatrics, University of CataniaCataniaItaly; 2 Department of Experimental and Clinical Pharmacology, University of CataniaCataniaItaly; 3 Department of Biochemistry, University of CataniaCataniaItaly

## Abstract

**Introduction:**

An epileptic child had been long treated with valproic acid and lamotrigine. After a few years of treatment, he manifested severe clinical signs of hypocalcemia. We retain that valproic acid could have caused such metabolic dysfunction.

**Case presentation:**

We report here the involvement of valproic acid in symptomatic hypocalcemia in an 11-year-old epileptic white patient in treatment with valproic acid and lamotrigine. During the treatment the patient developed hypocalcemia associated with high plasma levels of valproic acid, parathyroid hormone and alkaline phosphatase, indicating increased bone turnover. Plasma levels of Vitamin D were normal. Plasma calcium values significantly correlated with valproic acid haematic levels; reduction of valproic acid dose was accompanied by prompt normalization of calcemia.

The specific mechanism through which valproic acid causes hypocalcemia is unknown, although the relationship between valproic acid dose and haematic levels of calcium appears very likely.

**Conclusions:**

It seems necessary, during long term therapy with valproic acid, to monitor plasma calcium and alkaline phosphatase plasma levels. Also, these patients should undergo treatment and perhaps prescribe vitamin D and calcium treatment.

## Introduction

Hypocalcemia is defined as low calcium levels in the blood, usually taken as less than 8.5 mg/dL. A growing body of evidence suggests a relationship between this condition and long-term anticonvulsant therapy. Although this association has been initially confirmed only for those anti-epileptic drugs (AEDs) inducing the cytochrome P450 enzyme system due to the defective vitamin D metabolism and consequent reduction of intestinal absorption of calcium (i.e. phenobarbital, phenytoine, carbamazepine, primidone), more recent studies highlight the possible involvement of other anticonvulsant drugs, such as valproic acid. The mechanism involved in such alteration is still matter of open debate [1,2].

We herein report the case of an 11-year-old white, epileptic patient treated with valproic acid and lamotrigine who developed symptomatic hypocalcemia.

## Case presentation

We report here a case of an 11-year-old male white patient. The patient was first observed at the age of 6 months, with a 1-month history of infantile spasms and multiple malformations. Treatment with vigabatrin (VGB) was initiated and maintained for two years, when felbamate was associated for persisting Lennox-Gastaut-type seizures. Felbamate was withdrawn a few weeks later due to severe irritability.

At the age of 3 years, a number of metabolic, neuroradiologic and other investigations (arylsulfatase A, sialic acid, long chain fatty acids, urinary organic acid, Bratton-Marshall test, oligosaccharides, amino acids, hand X-ray, electromyography, nerve conduction velocity and karyotype) were performed and all tests were shown to be within normal ranges. The child was discharged with a diagnosis of Multiple Congenital Anomalies/Mental Retardation.

At the age of six years, due to the risk of adverse ocular effects of VGB, this drug was gradually replaced with valproic Acid (VPA). Several long-lasting seizure crises were observed during the early period of this regimen, followed by further crises of shorter duration in the following months. Therapy was therefore changed to a combination of Slow-release valproic acid (Depakin Chrono®) 825 mg/day and lamotrigine (Lamictal®) 25 mg twice daily, with complete control of seizure crises.

In 2006, at the age of 11 years, due to clinical signs of hypocalcemia (positive Trusseau sign, tetany, focal numbness, muscle spasms, laryngospasm and prolonged QT-c) the patient was hospitalized in the Pediatric Department of the University of Catania, Catania, Italy. Physical examination revealed macrocephaly, high-grade mental retardation, dyschromic and large teeth, a few café-au-lait spots on the right shoulder and leg, and right hemiparesis. The patient pronounced only some syllables, walked only if supported, and the patellar reflex was significantly augmented. Motor stereotypes of the hand and head were present, with absence of sphincter control. His dietary history did not reveal any abnormalities. Molecular investigations for Angelman Syndrome, ATRX syndrome, and FG syndrome were negative.

At this time, hypocalcemia (calcium 6.6 mg/dl, n.v. 8.5-10.5) and high levels of valproic acid (110 μg/ml, n.v. 40-100) were found in the blood, the remainder of the laboratory investigations were normal. Treatment with calcium and vitamin D was started and serum calcium was rapidly normalized.

In January 2007, the child was readmitted to the Department because, after a whithdraw of the calcium and vitamin D treatment, a new reduction of calcemia was noticed, albeit without clinical manifestations. Laboratory tests showed hypocalcemia (6.2 mg/dl), associated with high levels of VPA (106.00 µg/ml) and of PTH (289.00 pg/ml, n.v. 5-72) with normal levels of vitamin D (23.8 ng/ml n.v. 10-120), albumin (4.0 g/l), and phosphate (4.8 mg/dl). The high levels of alkaline phosphatase indicated an increased bone turnover and a state of decreased bone mineral density. The dose of valproic acid was considerably reduced, whereas the dose of lamotrigine was not varied, leading to reduction of plasma valproic acid levels and paralleled by normalization of plasma PTH, calcium and ALP values. The patient was followed up for 8 months, and the levels of calcium, vitamin D, and VPA in the blood have been maintained in the normal range. According to the Naranjo ADR probability scale, valproate subsequent hypocalcemia was classified as highly probable (score = 9).

## Discussion

Anticonvulsant drugs have hypocalcemic effects [1-5], with mechanism that differ depending upon the class of drugs considered. The inductors of the P450 cytochrome enzymes diphenylhydantoin, carbamazepine, pyridostigmine are known to enhance vitamin D catabolism, with a consequent reduction of its biological effects. These drugs can also inhibit the cellular response to parathyroid hormone, as well as reduce the intestinal absorption of cations.

Valproic acid is not an enzymatic inducer, nor it interferes with Vitamin D metabolism. However its metabolites act as anions, thus, binding plasma calcium ions and for this reason may cause hypocalcemia [6]. Furthermore, valproic acid and its metabolites are osmotically active anions that contribute to the hyperosmolality and acidosis. Its effects are dose- and time-dependent. Treatment with valproic acid may produce long-term effects as reduction of the bone mass, with increased risk of fractures, an effect associated with high levels of plasma alkaline phosphatase. Such risk increases proportionally with the duration of the therapy [4-7,8], although other studies are discrepant with this findings [9].

At our knowledge, lamotrigine does not cause hypocalcemia, nor it causes increased levels of markers of bone turnover in the blood, suggesting that this agent exerts no impact on bone mass even during long lasting treatments [2,10-12].

Our patient presented hypocalcemic convulsions after a 6 years treatment with both valproic acid and lamotrigine. The level of calcium was low (6.2 mg/dL); at the same time, plasma levels of valproic acid, parathyroid hormone and alkaline phosphatase were elevated, whereas plasma phosphate was normal (4.8 mg/dL). Apparently, dietary calcium intake was appropriate, so to exclude that hypocalcemia could originate from low calcium diet. Moreover, the child, although walking only if sustained, is being mobilized through appropriate physical exercises and physiotherapy, thus excluding another possible cause of hypocalcemia, immobilization.

Finally, the long-term therapy with valproic acid, as well as a diminished catabolic capability for the drug, could well account for hypocalcemia observed in this patient. In fact, clinical symptoms became milder and eventually disappeared concomitantly to the reduction of the dose of valproic acid. As a consequence of such dose reduction, we also observed normalization of plasma levels of parathyroid hormone and alkaline phosphatase.

Our assessed experience on epileptic pediatric patients using long-term treatment with valproic acid suggests that it is plausible that hypocalcemia could be related with the use of high doses of valproic acid.

Children long treated with high doses of valproic acid should be monitored for plasma calcium and alkaline phosphatase levels on a regular basis, in order that special care may be given to those patients who display drug metabolism-related disorders. Moreover, vitamin D and calcium treatment could be appropriately prescribed during long term therapy with valproic acid.

## Abbreviations

AED: anti-epileptic drugs
VGB: Vigabatrin
VPA: Valproic acid
